# Epidemiology and phylogeny of *Haemonchus contortus* through internal transcribed spacer 2 gene in small ruminants

**DOI:** 10.3389/fvets.2024.1380203

**Published:** 2024-04-09

**Authors:** Nisar Ahmad, Saeed A. Khan, Hafiz A. Majid, Rehman Ali, Riaz Ullah, Ahmed Bari, Noor Ul Akbar, Abdul Majid

**Affiliations:** ^1^Aquatic Eco-Health Group, Fujian Key Laboratory of Watershed Ecology, Key Laboratory of Urban Environment and Health, Institute of Urban Environment, Chinese Academy of Sciences, Xiamen, China; ^2^University of Chinese Academy of Sciences, Beijing, China; ^3^Department of Zoology, Kohat University of Science and Technology, Kohat, Pakistan; ^4^Department of Pharmacy, Kohat University of Science and Technology, Kohat, Pakistan; ^5^Livestock and Dairy Development (Research Wing), Kohat, Pakistan; ^6^Department of Pharmacognosy, College of Pharmacy, King Saud University, Riyadh, Saudi Arabia; ^7^Department of Pharmaceutical Chemistry, College of Pharmacy, King Saud University Riyadh, Riyadh, Saudi Arabia

**Keywords:** *Haemonchus contortus*, prevalence, small ruminants, ITS-2, PCR, phylogenetic analysis, epidemiology, phylogeny

## Abstract

**Introduction:**

*Haemonchus contortus* (*H. contortus*) is a blood-feeding nematode causing infectious disease haemonchosis in small ruminants of tropical and subtropical regions around the world. This study aimed to explore the prevalence and phylogeny of *H. contortus* in small ruminants using the internal transcribed spacer-2 (ITS-2) gene. In addition, a comprehensive review of the available literature on the status of *H. contortus* in Pakistan was conducted.

**Methods:**

Fecal samples were collected from sheep and goats (*n* = 180). Microscopically positive samples were subjected to DNA extraction followed by PCR using species-specific primers.

**Results:**

The overall prevalence of *H. contortus* was 25.55% in small ruminants. The prevalence of *H. contortus* was significantly associated with months and area. The highest occurrence of haemonchosis was documented in July (38.70%), whereas the lowest occurred in December (11.11%), with significant difference. The prevalence was highest in the Ghamkol camp (29.4%) and lowest in the arid zone of the Small Ruminant Research Institute (17.5%) (*p* = 0.01). The results of the systematic review revealed the highest prevalence of haemonchosis (34.4%) in Khyber Pakhtunkhwa (*p* = 0.001).

**Discussion:**

Phylogenetic analysis revealed a close relationship between *H. contortus* and isolates from Asia (China, India, Iran, Bangladesh, Malaysia, and Mongolia) and European countries (Italy and the United Kingdom). It has been concluded that *H. contortus* is prevalent in small ruminants of Kohat district and all over Pakistan, which could be a potential threat to food-producing animals, farmers, dairy, and the meat industry. Phylogenetic analysis indicates that *H. contortus* isolates share close phylogenetic relationships with species from Asia and Europe.

## 1 Introduction

*Haemonchus contortus* (*H. contortus*) is a parasitic worm causing haemonchosis in ruminants of tropical and subtropical climates around the globe (1, 2). Adult worms suck blood from the abomasum of goats and sheep, triggering edema, anemia, diarrhea, and sometimes death (3). Gastrointestinal parasites are abundant, and a single female is capable of producing up to 10,000 eggs each day (4). Eggs are excreted in host feces and dispersed into infective L3s on pastures, where they infect new hosts when consumed, and in turn infect millions of sheep and goats throughout the world. *Haemonchus* infects a wide variety of hosts and quickly becomes resistant to most of the anthelminthic drugs to contain it (5). It causes substantial economic losses due to the cost of anthelmintic medications, body weight loss, decrease in milk, meat, wool, and overall growth, and sometimes causes death to infected animals, adversely affecting livestock production (6).

*Haemonchus* is the most economically important blood-feeding parasite in grazing ruminants worldwide (7), with ancestral roots in sub-Saharan Africa, where numerous species of native artiodactyl hosts exist (8). Three known sympatric species of *Haemonchus* infect ruminants across Asia. A greater number of *H. contortus*, *H. longistipes*, and *H. placei* have been documented due to the international movement of domesticated animals (7, 8). *H. contortus* is primarily a small ruminant parasite; *H. longistipes* is most frequently reported in camels; and *H. placei* infects cattle. In addition, *H. similis*, the fourth sympatric species, has been found to infect Latin American cattle (9).

Helminth resistance to numerous anthelmintic medications is rapidly growing, causing significant public health problems. In the near future, controlling some parasites with current anthelminthic medications like oxfendazole, levamisole, and ivermectin may become more challenging (10). *H. contortus* has evolved different strategies to evade host immune response during infection (12, 13). Parasitic nematodes cause a long-term infection in the host, generally with just a modest inflammatory response. This is attributed to the release of complex excretory/secretory protein (ESP) mixtures into host tissues that interfere with host signaling mechanisms and immune homeostasis. However, these molecular mechanisms are yet to be explored and need further in-depth research. Therefore, to control parasitic infections researchers are actively exploring alternative methods such as nanovaccine (14, 15), nanoparticle (16), and plant extracts have shown promising anti-parasitic activity to counter anthelminthic resistance in *H. contortus* (17–19).

Pakistan is mainly an agricultural country contributing 22.9% to the Gross Domestic Product (GDP), where livestock account for approximately 62.68% of agriculture and 14.36% of the national GDP (20). *H. contortus* infestation has become one of the biggest problems of sheep and goat husbandry in Pakistan. Aside from the lack of meat due to mortality and lower growth in small ruminants, there is an exceedingly high proclivity to gain immunity against anthelmintic medications, and a proven model for researching the genetics and population structure of resistant strains (9).

Environmental and geographical constraints, population growth, and living conditions are a few of the variables that alter a population’s genetic makeup (21). *Haemonchus* is favored by a high rate of gene flow across populations, providing an opportunity for the dissemination of genes conferring resistance to anthelmintics (22). Parasitic nematode ecology, epidemiology, and evolution can be better understood by studying genetic diversity and genetic relationships using phylogenetic trees (23). In addition, precise identification and genetic characterization are essential for a valid diagnosis and efficacy in the control programs of parasitic nematodes (3). The ribosomal *ITS-2* gene is one of the most variable nuclear loci, and its high rate of evolution can be used to achieve intraspecific variation among *H. contortus* populations (24). This nematode marker has been used in species differentiation and genetic variability studies. Interpretation of the inherited differences within and among *H. contortus* species may promote a better understanding of transmission patterns and the establishment of a control strategy (22).

Controlling haemonchosis is essential to recouping losses, since it significantly harms Pakistan’s small ruminant industry. To ensure effective and reliable parasite control, it is necessary to have a thorough understanding of the current epidemiological parameters influencing the distribution of disease. Numerous studies have been conducted on *H. contortus* in ruminants around Pakistan, as shown in Table 1; however, no microscopic, molecular, or phylogenetic analysis study has been found in the province of Khyber Pakhtunkhwa, particularly district Kohat. Therefore, the current study was designed to investigate epidemiology and phylogeny of *H. contortus* species in small ruminants with a systematic review of Pakistan’s previously published studies on ruminants to provide baseline information. The paucity of molecular data on *Haemonchus* in the small ruminant population of Kohat district was the main impetus to carry out this investigation. The current study will advance our knowledge of Pakistan’s haemonchosis epidemiology, including its genesis, transmission patterns, and population structure, which might direct government actions to halt the spread of the disease.

**Table 1:** Studies on *H. contortus* prevalence in Pakistani ruminants.

Authors	Host species	Sampling area	Sample type	Study Method	No. of samples	Positive Samples	Prevalence (%)	Random sampling/not	Sampling method clear/not	Sampling method detailed/not	No. of samples>80 or not	Risk factor > 3	Score	Study quality
Ali et al. (25)	Sheep	Kohat	Blood and Fecal	Direct microscopy	150	39	26	No	Yes	No	Yes	Yes	2	Average
	Goat				150	17	11.33							
Ruhoollah et al. (26)	Sheep	Dir Upper	Fecal	Direct smear method	184	40	21.73	Yes	No	No	Yes	Yes	3	Average
	Goat				131	31	28.7							
Bibi et al. (27)	Sheep	Lahore, Multan, Mandi Bahauddin & Haripur	Worm	Microscopy	100	55	55	No	Yes	No	Yes	Yes	3	Average
	Goat				100	50	50							
	Cattle				100	35	35							
Qasim et al. (28)	Sheep	Lodhran	Fecal	Direct smear & EPG method	323	97	30.03	Yes	Yes	Yes	Yes	Yes	5	Good
	Goat	Lodhran	Fecal	Direct smear & EPG method	323	81	25.07	Yes	Yes	Yes	Yes	Yes	5	Good
Jamal et al. (29)	Markhor	Chitral	Fecal	Simple direct method	25	10	40	Yes	Yes	No	No	No	2	Average
Jamil et al. (30)	Sheep	Dera Ismail Khan	Fecal	Direct microscopy, floatation, sedimentation & McMaster	300	120	40	No	Yes	Yes	Yes	Yes	4	Good
Lashari and Tasawar (31)	Sheep	Dera Ghazi khan, Multan & Khanewal	Fecal	Direct microscopy, floatation & sedimentation	523	34	6.5	Yes	Yes	No	Yes	Yes	4	Good
Raza et al. (32)	Sheep	Bahawalpur, Rahim Yar Khan & Bahawalnagar	Fecal	Floatation & sedimentation	500	69	13.8	Yes	Yes	No	Yes	Yes	4	Good
	Goat				500	64	12.8							
Khalid et al. (33)	Sheep	Lahore	Fecal	Microscopy, floatation, sedimentation & McMaster	240	71	29.58	No	Yes	No	Yes	Yes	4	Good
	Goat				240	66	27.5							
Razzaq et al. (34)	Sheep	Loralai	Fecal	McMaster	1,200	125	10.42	No	No	No	Yes	Yes	2	Average
Ayaz et al. (35)	Goat	Muzaffargarh	Fecal	Direct microscopy, floatation & sedimentation	100	20	20	Yes	No	No	Yes	Yes	3	Average
Ullah et al. (36)	Sheep	Peshawar	Fecal	Microscopic & floatation	356	190	53.37	No	Yes	No	Yes	Yes	3	Average
Akhter et al. (37)	Goat	Hyderabad	Fecal	Direct smear microscopy	1,065	156	14.65	Yes	Yes	No	Yes	No	3	Average
Tasawar et al. (38)	Sheep	Khanewal	Fecal	McMaster	333	259	77.7	No	Yes	No	Yes	Yes	3	Average
Khan et al. (39)	Sheep	Toba Tak Singh	Fecal	Floatation	840	193	22.98	Yes	Yes	Yes	Yes	Yes	5	Good
	Goat				660	162	24.55							
	Cattle				1,140	152	13.33							
	Buffaloe				1,140	163	14.3							
Gadahi et al. (40)	Sheep	Rawalpindi	Fecal	Direct microscopy, floatation & sedimentation	90	26	28.88	No	No	No	Yes	No	1	Below average
	Goat				310	199	64.19							
Raza et al. (41)	Sheep	Multan	Worm	Key identification	2,133	793	37.18	No	No	No	Yes	No	1	Below average
	Goat				2,607	811	31.1							
Ijaz et al. (42)	Goat	Lahore	Fecal	Direct Smear, floatation & McMaster	300	84	28	No	Yes	No	Yes	No	2	Average
Lateef et al. (43)	Sheep	Faisalabad	Worm	Microscopy & key identification	960	591	61.5	Yes	Yes	No	Yes	No	3	Average
Jabeen et al. (44)	Sheep	Lahore, Gujranwala, Faisalabad, Sargodha, Rawalpindi, Multan, D.G. Khan & Bahawalpur	Fecal	Microscopy	10,000	5,477	54.77	Yes	No	No	Yes	No	2	Average
Sajid et al. (45)	Sheep	Islamabad	Fecal	Floatation, & McMaster	62	54	87	No	No	No	No	No	0	Below average

## 2 Methodology

### 2.1 Ethical approval

The current study was approved from Kohat University of Science and Technology Kohat ethical approval committee for sample collection from animals. Written informed consent has been granted by the owners for their animal’s participation in the current study.

### 2.2 Study area and sample collection

**Figure 1:**
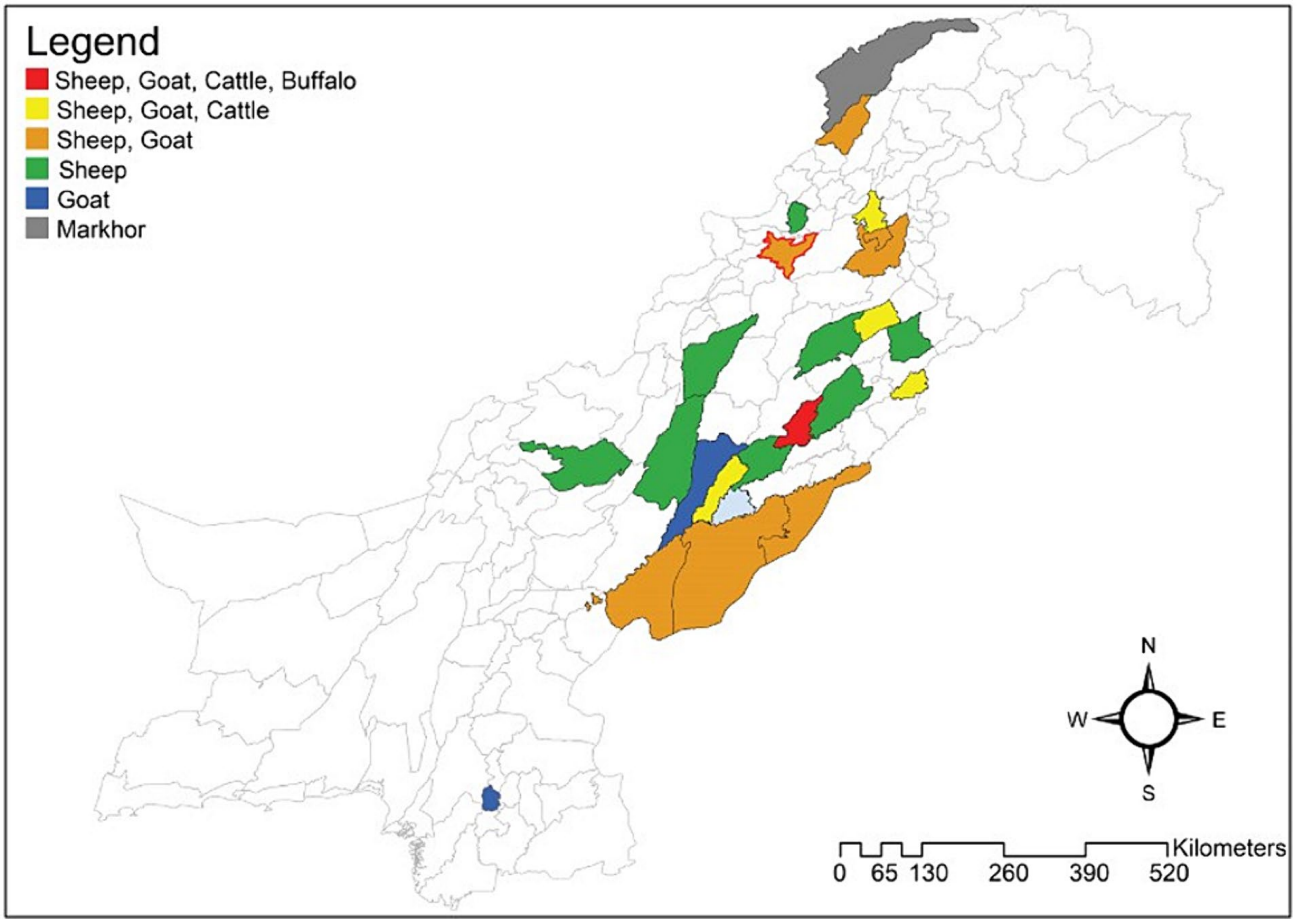
Pakistani map with colored areas displays epidemiological studies performed on *Haemonchus contortus* in different districts. 1) Red indicates studies performed on sheep, goats, cattle, and buffaloes in that area; 2) sheep, goat, and cattle investigations are shown in yellow. 3) The golden hue indicates that both sheep and goats were examined for *H. contortus* in certain areas. 4) Studies on sheep are indicated in green. 5) Studies involving goats are shown in blue. 6) Studies involving only markhor are displayed in gray, and 7) Red outline on golden hue color shows the current study sampling area district Kohat. (Drawn using the software “ArcGIS” (https://desktop.arcgis.com/en/)).

The present study was done at Kohat, Khyber Pakhtunkhwa. It lies 489 m above sea level between 32° 47′ and 33° 53′ north latitude and 70° 34′ and 72° 17′ east longitude (Figure 1). Approximately 5 g of feces was directly obtained from the rectum of 180 sheep and goats (90 each) under strict aseptic conditions in the study area. All information, including the animal type, sex, age, location, and collection month, was recorded on the mandated proforma for each sheep and goat. The Molecular Parasitology and Virology Laboratory, Department of Zoology, KUST, received appropriately labeled samples in cold boxes. Prior to further processing, the samples were preserved at −20°C.

### 2.3 Slides preparation & microscopic examination

Three grams of feces were added to container 1 with 50 mL of PBS dispensed into the same container, and a tongue blade was used to mix the feces with the PBS solution. The fecal suspension was transferred from the jar to container 2, and finally to the test tube using a double layer of cheesecloth. A convex meniscus formed on top of the test tube after the solution was^1^ gently tapped off. A delicate coverslip was set atop the test tube and left undisturbed for 20 min. Twenty minutes later, the coverslip and drop of fluid attached to it were carefully removed from the test tube and placed on a clean slide. For microscopic positive samples, the tube was filled with 2 mL of PBS and spun in a microcentrifuge for 5 min at 3000 rpm to rinse the eggs. After the supernatant was removed, the rinsing procedure was repeated. Once the supernatant was removed, 300 𝜇l of the ova in the pellet was collected for genome extraction (46). Coverslips adhering to the slide were stained with iodine and examined for eggs and larvae using a light compound microscope under 10X and 40X objective lens, according to Ebrahim (47).

### 2.4 DNA extraction, amplification and gel electrophoresis

*Haemonchus* positive samples were extracted using the “QIAamp Fast DNA Stool Mini Kit” (Qiagen GmbH, Hilden, Germany). The manufacturer’s protocol was used to extract the complete genomic DNA. Amplification of the prepared DNA was carried out as described by Hussain et al. (9). The ITS2 gene was amplified using NC1F (5’-ACGTCTGGTTCAGGGTTGTT-3′) and NC1R (5’-TTAGTTTCTTTTCCTCCGCT-3′) with an amplicon size of 350 bp (23). To achieve the necessary gene amplification, a thermal cycler was utilized with a 25 𝜇l PCR reaction volume comprising of 13 𝜇l PCR Master mix (dNTPs, MgCl2, Taq DNA polymerase), 1 𝜇l forward and reverse primers, 5 𝜇l PCR water (dH2O), and 5 𝜇l extracted DNA. The initial temperature was 96°C for 7 min, followed by 40 cycles of 95°C for 45 s, 57.5°C for 45 s, and 72°C for 45 s, with a final extension temperature of 72°C for 7 min. A 1.5% agarose gel was used for DNA resolution. Bands (Fermentas, United States) were compared to a 2000 bp DNA ladder marker (23). A gel documentation device was used to resolve the bands under UV illumination.

### 2.5 Searching strategy

Adherence to Preferred Reporting Items for Systematic Review and Meta-analysis (PRISMA) was maintained throughout the execution of this review on articles related to *H. contortus* epidemiology in ruminants across Pakistan (10). Research articles published in English were retrieved from different databases, including ISI Web of Science, Mendeley, PubMed, ScienceDirect, EBSCHO, and Google Scholar. Databases were searched for articles using keywords such as prevalence, epidemiology, infection rate/infestation rate, *Haemonchus contortus*, *Haemonchus*, sheep, goat, ruminants, and Pakistan. Boolean operators “AND” and “OR” were also used to retrieve articles (“Prevalence” OR “epidemiology” OR “infection rate” OR “infected” AND “*Haemonchus contortus*” AND “Pakistan” AND “ruminants” OR “sheep” OR “goat”) on the *H. contortus* distribution rate across Pakistan from January 2000 till July 2023. The article references and PDFs were downloaded to a folder created using Mendeley reference manager software for the current study. Duplicate studies were removed from the folder after checking the title and references, followed by irrelevant article removal due to the non-availability of data in the abstract.

### 2.6 Inclusion and exclusion criteria

We checked the article titles and abstracts to determine whether they had any data on the prevalence of *H. contortus* in Pakistan. The inclusion criteria were as follows: i) articles written in English, ii) articles containing *H. contortus* distribution in ruminants, and iii) articles published between January 2000 and July 2023. However, we excluded reviews, duplicate articles, studies dealing with other species of *Haemonchus* prevalence data, articles involving two species without confirmation of a single species outcome, publications with vague data such as trichostrongylid/strongyles infection rate, or sources of samples without complete text.

### 2.7 Quality assessment

The current systematic review included studies that were evaluated using the following criteria to ascertain quality scores: (a) random sampling/not, (b) sampling method clear/not, (c) sampling method detailed/not, (d) number of samples ≥80/not, and (e) ≥3 risk factors. By calculating the aforementioned answers, each study was given a score; a score of 0 for “no” and a score of 1 was given for “yes,” aggregating a total score of 5. Studies with a score of 0–1, 2–3, and 4–5 were classified as below average, average, and good quality, respectively.

### 2.8 Study selection and data extraction

**Figure 2:**
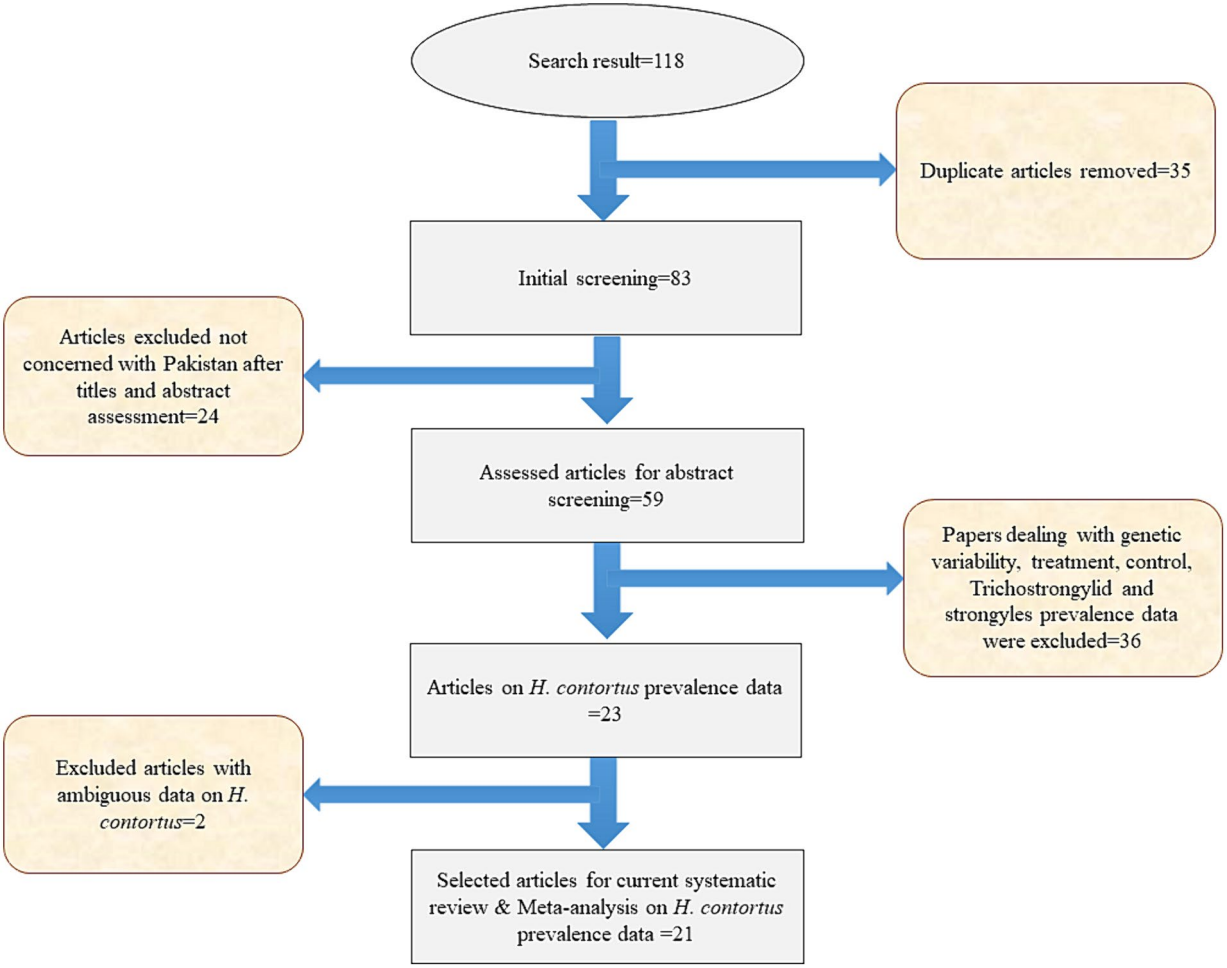
Article screening and selection process used for conducting current systematic review.

To ensure that no papers were overlooked, two writers (NA and RA) separately searched the English databases for titles and abstracts of articles that met the inclusion criteria. The eligibility of the study for inclusion was determined by the same writers who independently reviewed the entire text. Potentially eligible studies were excluded if (i) the prevalence data results were not provided in terms of the number of samples and (ii) the dataset was ambiguously presented based on ruminants. Another author (AM) made the ultimate choice following a debate in which the two writers could not reach an agreement. Finally, the data were extracted by NA and RA, which included pertinent information about the initial author, publication year, host species, type of sample, method of study, number of examined samples, positive sample, prevalence rate, random sampling/not, clear sampling method/not, detailed sampling method/not, number of samples ≥80/not, and ≥ 3 risk factors studies/not to calculate the score and quality of included studies using a pre-designed Microsoft Excel spreadsheet (Figure 2).

### 2.9 Sequencing and phylogenetic analysis

After amplicon confirmation through gel electrophoresis, 4 randomly selected amplified DNA products (two each sheep and goat samples) were shipped to Macrogen Inc. (Seoul, South Korea) for purification and sequencing. The chromatograms of the sequenced samples were interpreted, and noise was removed at the start and end of each sequence using BioEdit software. The resulting sequences were subjected to BLAST in NCBI^2^ for confirmation of *H. contortus*. After confirmation of *H. contortus*, the reference sequences were retrieved and transferred to Mega11 for further processing. Sequences were trimmed and aligned using Clustal *W*, followed by the inclusion of an outgroup in the software, and the maximum composite likelihood technique was used for phylogenetic analysis. The sequences from this study and those from the NCBI gene repository were used to construct a phylogenetic tree. Through the addition of a maximal composite probability parameter and 1,000 bootstrap replications, an evolutionary tree was constructed using the maximum composite likelihood approach. Nucleotide sequences reported in the present study are available in GenBank^™^, an NCBI database, under the accession numbers OK447878, OK481181, OM276841, and OM276825.

### 2.10 Data analysis

To determine the differences between the observed and expected data, numerous factors, including type of ruminant, age, sex, month, and distribution by locality, were analysed in relation to the prevalence of haemonchosis using Chi-squared and Fisher exact tests in R software. A *p*-value of less than 0.05 was used to determine the statistical significance of the findings (11).

## 3 Results

### 3.1 Epidemiology of *Haemonchus contortus* in small ruminants

**Figure 3:**
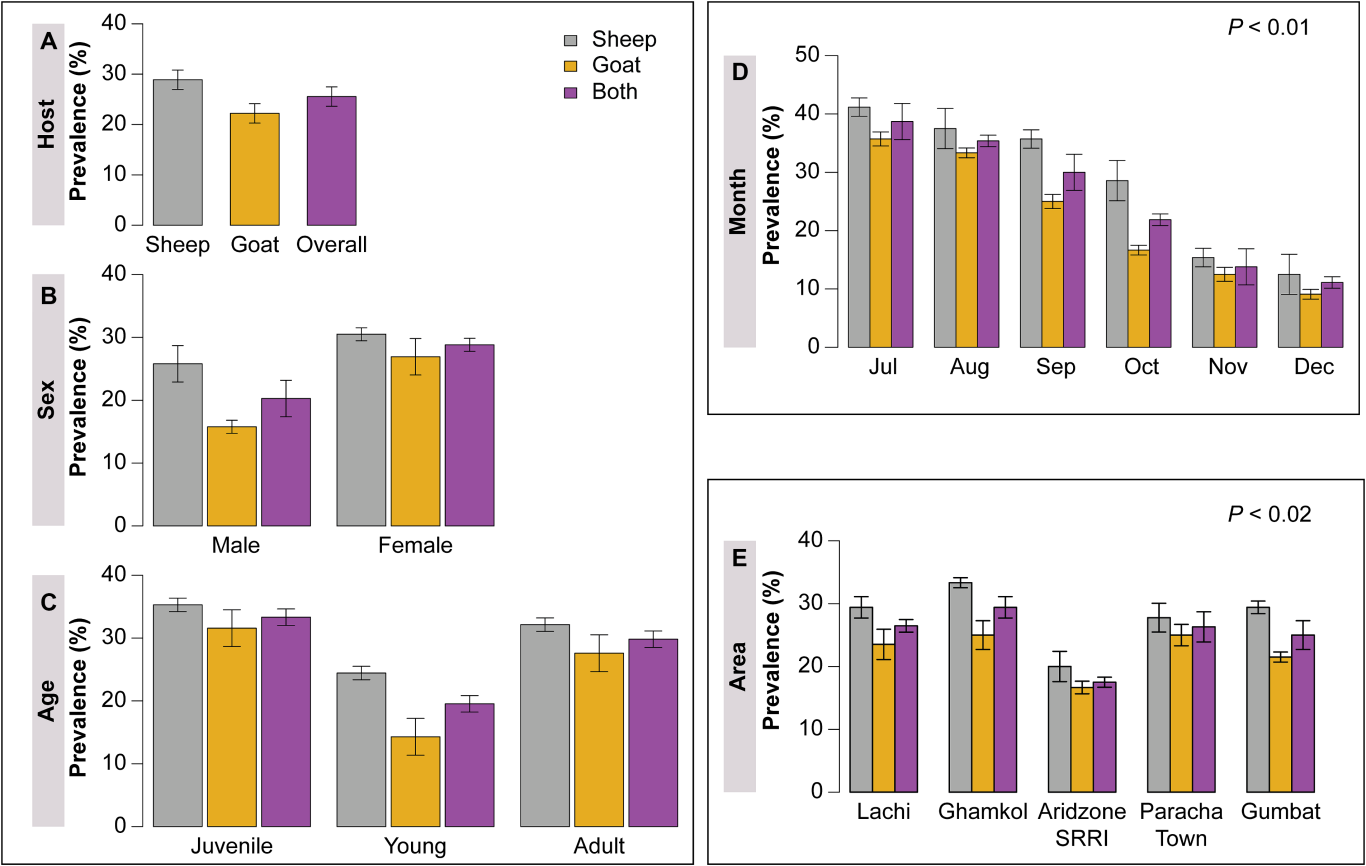
**(A)** Showing host based overall prevalence of *H. contortus*, **(B)** sex wise prevalence of sheep and goat, **(C)** *H. contortus* occurrence based on age group, **(D)** area wise prevalence, and **(E)** Month wise prevalence of *H. contortus* in sheep and goats.

A total of 180 fecal samples were collected from both sheep and goats of different age groups, sex, months, and area from July to December, 2021. Forty-six (46) small ruminants were found positive for haemonchosis. The documented overall prevalence of *H. contortus* in small ruminants was 25.55%. However, sheep was non-significantly more susceptible (28.89%) to haemonchosis in comparison to goats (22.22%) *p* > 0.05 (Figure 3A). Both goats and sheep were divided into male and female categories based on sex. The sex-wise infection of *H. contortus* recorded in the current research work was higher in female sheep and goats (28.82%) as compared to male sheep and goats (20.28%) *p* > 0.05 (Figure 3B).

Small ruminants were divided into three age groups: <1 year, 1–3 year, and > 3 years named as juvenile, young and adult, respectively. The highest prevalence rate was recorded in juvenile (33.33%) and adult age groups (29.82%), while the lowest was in the young age group (19.54%) however, the differences were non-significant *p* > 0.05 (Figure 3C). The present study was divided into 5 different localities of district Kohat named Lachi, Ghamkol Camp, Arid Zone Small Ruminant Research Institute (SRRI), Paracha Town, and Gumbat. The highest occurrence of *Haemonchus* was observed in Ghamkol camp (29.41%), while the lowest was in the Arid Zone (SRRI) (17.5%) of district Kohat *p* < 0.02 (Figure 3D). The current study was comprised of 6 months of sample data from July to December. The highest incidence of 38.70% of *H. contortus* was documented in the month of July, whereas the lowest was 11.11% in December *p* < 0.01 (Figure 3E).

### 3.2 Bibliographic search and quality assessment

We identified and added a total of 118 articles PDF with references from our searched databases using Mendeley Web Importer. We removed 35 duplicate articles from the Mendeley reference manager software dedicated folder for the current study. After removing duplicate articles, 83 articles remained. Primary screening of the title and abstract resulted in the exclusion of 24 papers not concerning with Pakistan; the remaining 59 articles were then chosen for full-text reading. In addition, 38 studies were removed according to our inclusion and exclusion parameters. Finally, 21 eligible papers based on *H. contortus* epidemiology in Pakistani ruminants were eventually included in the current systematic review, as shown in Table 1.

Out of 21 articles, the highest number of studies 13 (61.90%) were reported from Punjab, followed by Khyber Pakhtunkhwa 5(23.80%) and 1(4.76%) each from Baluchistan, Sindh, and Islamabad capital territory. The current systematic review consists of *Haemonchus contortus* studies on the ruminant population, which includes sheep, goats, cattle, buffalo, camels, and markhor. The most common types of samples used for *H. contortus* identification were fecal 17, followed by worm 3, and a single study collected both blood and fecal samples. Multiple methods were used for *H. contortus* evaluation, including direct smear microscopy, key identification, floatation, sedimentation, McMaster, and the Eggs per gram method. The most common evaluation method was direct microscopy, while the least common was key identification, as shown in Table 1. Based on risk factors 12 studies fall in the average category, followed by 6 in the good category and 3 in the below-average category.

**Figure 4:**
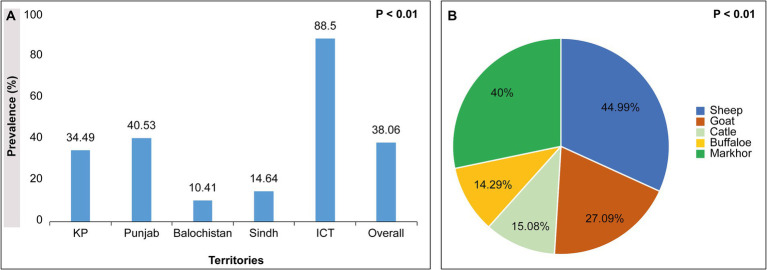
**(A)** showing territory wise prevalence of *H. contortus*; **(B)** showing animal wise prevalence of *H. contortus* in ruminants across Pakistan.

The 21 selected Pakistani studies on ruminants show a 38.06% overall prevalence rate of *H. contortus* across Pakistan. Studies performed in all 5 regions of Pakistan, namely Khyber Pakhtunkhwa, Punjab, Baluchistan, Sindh, and Islamabad capital territory area, accounted for 34.49, 40.53, 10.41, 14.64, and 88.5%, respectively, *p* < 0.001. Based on the type of animal, sheep, goat, cattle, buffalo, and markhor show 44.99, 27.09, 15.08, 14.29, and 40% *H. contortus* with significant difference *p* < 0.001 respectively, Figure 4.

### 3.3 Phylogenetic tree

Followed by DNA extraction and amplification of the ITS-2 gene, a PCR product of approximately 350 bp was found under UV light. The top hits and highly similar sequences were retrieved for downstream phylogenetic tree construction. A dendrogram was constructed for the ITS-2 gene of *Haemonchus* spp. and other related nematode genera, including Marshallagia and Trichostrongylus. The dendrogram is comprised of 4 sequences from Kohat and 24 published sequences of different geographical localities, including Pakistan, China, India, Malaysia, Iran, Bangladesh, Thailand, Mongolia, Yemen, Italy, Denmark, and Japan, among others.

**Figure 5:**
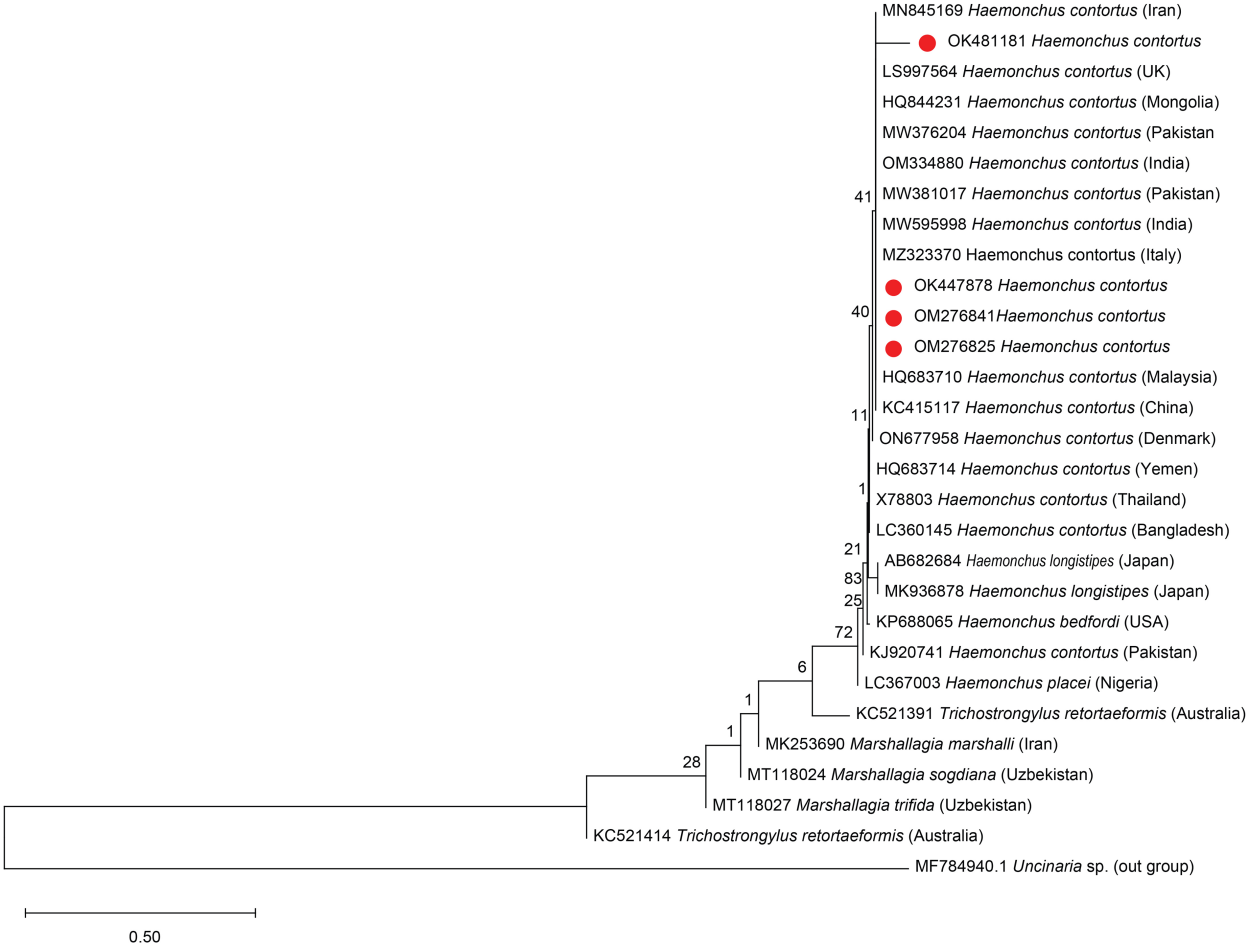
Phylogenetic tree of *H. contortus* in sheep and goat using ITS2 marker. The evolutionary history was inferred using the Neighbor-Joining method (48). The optimal tree is shown. The percentage of replicate trees in which the associated taxa clustered together in the bootstrap test (1,000 replicates) are shown next to the branches (49). The tree is drawn to scale, with branch lengths in the same units as those of the evolutionary distances used to infer the phylogenetic tree. The evolutionary distances were computed using the Maximum Composite Likelihood method (50) and are in the units of the number of base substitutions per site. This analysis involved 29 nucleotide sequences. All positions with less than 95% site coverage were eliminated, i.e., fewer than 5% alignment gaps, missing data, and ambiguous bases were allowed at any position (partial deletion option). There were total of 88 positions in the final dataset. Evolutionary analyses were conducted in MEGA11 (51).

The phylogenetic analysis clustered *H. contortus* into a major clade with different subclades (Figure 5). *H. contortus* is grouped separately from other related species, i.e., *H. bedfordi*, *H. longistipes*, and *H. placei*. The *H. contortus* of the current study was closely related to the *H. contortus* of the neighboring Asian countries, including Iran, India, China, Mongolia, and Malaysia. This subclade also comprised a sequence from district Jhang of Pakistan, United Kingdom, Italy, and Mongolia. Surprisingly, our study’s neighbor joining dendrogram (Figure 5) showed that the isolates under investigation were quite closely related to those from Italy and the United Kingdom.

## 4 Discussion

*Haemonchus contortus* is an efficient blood feeding parasite that causes significant economic losses to the livestock sector via decreased body weight, milk, meat, and wool production, in addition to other forms of harm and often the death of afflicted animals. Considering the significance of haemonchosis, the current study was carried out with the goal of determining the prevalence and phylogenetic analysis of *H. contortus* using microscopic and genetic marker ITS2 in small ruminants of district Kohat with a systematic review of Pakistan *H. contortus* prevalence studies to provide baseline information.

Our findings of 27.55% *H. contortus* in sheep and goats of district Kohat are lower than the total 38.06% prevalence rate in 21 selected studies across Pakistani ruminants, with 34.49% *Haemonchus* occurrence in our study area province Khyber Pakhtunkhwa, as shown in Table 1. Earlier, several studies indicated varying prevalence rates of haemonchosis in the small ruminants of Pakistan, with numbers ranging from 6 to 88%, as shown in Table 1. Researchers across the globe observed 3–90% of haemonchosis infection rate in small ruminants (52–59). Possible explanations for the observed variation in prevalence include differences in sample size, seasonality, environmental variability, and management practices in the research region. Several variables, including management practices, grazing patterns (such as mixed herds of male, female, juvenile, and adult animals), farmers incomes and education levels, and the irrational use of anthelmintics, might affect the parasite population density (31, 40).

The total recorded sex-wise prevalence of haemonchosis in small ruminants was greater in females (28.82%) than males (20.28%) *p* > 0.05. Our findings are consistent with those of Brik et al. (59) and Raza et al. (41), who found a greater rate of haemonchosis in females (30.98 and 35.19%) than in males (15.63 and 31.80%), respectively. Whereas, the present research contradicts Nabi et al. (60), and Tassawar et al. (2010) as they reportedhigher prevalence rate of *H. contortus* in male as compared to female population. The increased prevalence of haemonchosis may be attributed to female sensitivity to parasitism as a result of reproductive stress and a weakened immune system (61).

The age-specific prevalence rate of haemonchosis in small ruminants showed that juvenile age group is most vulnerable to *H. contortus* (33.33%), followed by adult age group (29.82%), with the lowest infection rates found in young age group (19.54%) *p* > 0.05. Our findings concur with those of (38, 62–64), as they also noted a higher *H. contortus* infection rate in the younger and older age groups. Higher infection rates in the younger and older age groups could be associated with reduced immunity in these animals since most cases of haemonchosis afflict either immunocompromised adults or non-immune young animals (i.e., during the first grazing season) (65).

Month-wise observed prevalence rates of *H. contortus* in small ruminants show that July and August had the highest rates of infection (38.71 and 35.48%, respectively), followed by September (30%), whereas December had the lowest rate of infection (11.11%) *p* < 0.01. Our results are in agreement with (43, 66–69) as they also reported a higher infection rate in humid conditions. The elevated biotic potential of *H. contortus* infection contributes to its swift predominance at a time when environments on pastures are favorable for free-living phases to develop and survive (69). Similar to our finding, Durrani et al. (70) and Rizvi et al. (71) also reported a higher prevalence of haemonchosis in the months of July and August, with the authors explaining that the presence of moisture favored the growth of the larvae.

The highest haemonchosis was documented in the localities of Ghamkol camp (29.41%), while the lowest prevalence was recorded in Arid zone (SSRI) 17.5% with *p* < 0.02. Variations in the prevalence rate of haemonchosis in sheep and goats might be influenced by different factors, including the grazing behavior, economic status, education level of the farmers, differential management practices, natural resistance, nutrition, and anthelmintics used (72).

The current study result is consistent with previous investigations as we found a single *Haemonchus* specie, *H. contortus*, in both sheep and goats, showing close association with the same geographical region and neighboring countries, including Iran (73), Pakistan (Jhang) (74), India (OM334880, MW595998), China (22), Malaysia (24), and Mongolia (844231). However, our *H. contortus* samples from both sheep and goat yielded an unexpected finding in the neighbor joining dendrogram (Figure 5), showing a strong correlation with the isolates from Italy (75) and the United Kingdom (LS997564) of distinct European continent. Troell et al. (76) and Dey et al. (23) also reported similar findings, where isolates from Greece overlapped with the obtained isolates of Australia and Malaysian isolates were closely related to American isolates, respectively. Since there is no proof of direct animal migration across two continents, the cause of these occurrences is the introduction of parasite populations via imported animals of the same provenance (23).

The primary limitation of the current research work was our inability to target multiple districts of Khyber Pakhtunkhwa province for sampling and sequenced limited samples for *H. contortus* identification and genetic variability in sheep and goats of district Kohat owing to limited time of study.

## 5 Conclusion

We concluded that *H. contortus* is prevalent across Pakistan, particularly in district Kohat and ITS2 genetic marker confirms our microscopic and molecular identification. The incidence of *H. contortus* is correlated with area and seasonal groups. Phylogenetic analysis shows a close association with Asian and European (Italian and UK) isolates. *Haemonchus contortus* must be further studied in unexplored areas in future studies, and other genes should be targeted for more diverse results.

## Data availability statement

The datasets presented in this study can be found in online repositories. The names of the repository/repositories and accession number(s) can be found in the article/supplementary material.

## Ethics statement

The animal studies were approved by the Kohat University of Science and Technology Kohat ethical approval committee for sample collection from animals. The studies were conducted in accordance with the local legislation and institutional requirements. Written informed consent was obtained from the owners for the participation of their animals in this study.

## Author contributions

NA: Conceptualization, Data curation, Formal analysis, Investigation, Methodology, Software, Visualization, Writing – original draft, Writing – review & editing. SK: Funding acquisition, Resources, Supervision, Writing – review & editing. HM: Investigation, Supervision, Writing – review & editing. RA: Data curation, Investigation, Software, Writing – review & editing. RU: Funding acquisition, Methodology, Writing – review & editing. AB: Funding acquisition, Writing – review & editing. NUA: Investigation, Writing – review & editing. AM: conceptualization, Resources, Supervision, Validation, Writing – review & editing.

## Footnotes

^1^ https://www.rvc.ac.uk/review/parasitology/Flotation/Simple_flotation/Purpose.html

^2^ http://www.ncbi.nlm.nih.gov

## References

[1] Wen Z Aleem MT Aimulajiang K Chen C Liang M Song X. The GT1-TPS structural domain protein from *Haemonchus contortus* could be suppressive antigen of goat PBMCs. Front Immunol. (2022) 12:787091. doi: 10.3389/fimmu.2021.787091, PMID: 35058927 PMC8764253

[2] Wen ZH Xie XR Aleem MT Aimulajiang K Chen C Liang M. In vitro characterization of *Haemonchus contortus* trehalose-6-phosphate phosphatase and its immunomodulatory effects on peripheral blood mononuclear cells (PBMCs). Parasit Vectors. (2021) 14:611. doi: 10.1186/s13071-021-05115-4 34930417 PMC8685816

[3] Gasser RB Bott NJ Chilton NB Hunt P Beveridge I. Toward practical, DNA-based diagnostic methods for parasitic nematodes of livestock—bionomic and biotechnological implications. Biotechnol Adv. (2008) 26:325–34. doi: 10.1016/j.biotechadv.2008.03.003, PMID: 18502076

[4] Prichard R. Genetic variability following selection of *Haemonchus contortus* with anthelmintics. Trends Parasitol. (2001) 17:445–53. doi: 10.1016/S1471-4922(01)01983-3, PMID: 11530357

[5] Qamar W Alkheraije KA. Anthelmintic resistance in *Haemonchus contortus* of sheep and goats from Asia--a review of in vitro and in vivo studies. Pak Vet J. (2023):43, 376–387. doi: 10.29261/pakvetj/2023.088

[6] Nazish A Fozia Khattak B Ali Khan T Ahmad I Ullah R. Antinematode activity of abomasum bacterial culture filtrates against *Haemonchus contortus* in small ruminants. Animals (Basel). (2021) 11:1843. doi: 10.3390/ani11061843, PMID: 34205748 PMC8235536

[7] Gilleard JS. *Haemonchus contortus* as a paradigm and model to study anthelmintic drug resistance. Parasitology. (2013) 140:1506–22. doi: 10.1017/S0031182013001145, PMID: 23998513

[8] Hoberg EP Lichtenfels JR Gibbons L. Phylogeny for species of Haemonchus (Nematoda: Trichostrongyloidea): considerations of their evolutionary history and global biogeography among Camelidae and Pecora (Artiodactyla). J Parasitol. (2004) 90:1085–102. doi: 10.1645/GE-3309, PMID: 15562609

[9] Hussain T Periasamy K Nadeem A Babar ME Pichler R Diallo A. Sympatric species distribution, genetic diversity and population structure of Haemonchus isolates from domestic ruminants in Pakistan. Vet Parasitol. (2014) 206:188–99. doi: 10.1016/j.vetpar.2014.10.026, PMID: 25468018

[10] Ali R Ahmad N Mussarat S Majid A Alnomasy SF Khan SN. Nanoparticles as alternatives for the control of *Haemonchus contortus*: a systematic approach to unveil new anti-haemonchiasis agents. Front Vet Sci. (2021) 8:789977. doi: 10.3389/fvets.2021.789977, PMID: 34966814 PMC8710572

[11] R Core Team (2022). R: a language and environment for statistical computing. R Foundation for Statistical Computing, Vienna, Austria. Available at: https://www.R-project.org/

[12] Liang M Lu M Aleem MT Zhang Y Wang M Wen Z. Identification of excretory and secretory proteins from *Haemonchus contortus* inducing a Th9 immune response in goats. Vet Res. (2022) 53:36. doi: 10.1186/s13567-022-01055-8, PMID: 35597967 PMC9123704

[13] Wen Z Zhang Y Feng J Aimulajiang K Aleem MT Lu M. Excretory/secretory proteins inhibit host immune responses by downregulating the TLR4/NF-κB/MAPKs signaling pathway: a possible mechanism of immune evasion in parasitic nematode *Haemonchus contortus*. Front Immunol. (2022) 13:1013159. doi: 10.3389/fimmu.2022.1013159, PMID: 36238295 PMC9551057

[14] Wang Q Muhammad TA Muhammad WH Muhammad AM Muhammad H Yan R. Hepatocellular carcinoma-associated antigen 59 and ADP-ribosylation factor 1 with poly (lactic-co-glycolic acid): a promising candidate as nanovaccine against haemonchosis. Microb Pathog. (2022) 168:105614. doi: 10.1016/j.micpath.2022.105614, PMID: 35662672

[15] Wang Q Muhammad TA Muhammad WH Muhammad AM Muhammad H Yan R. *Haemonchus contortus* hepatocellular carcinoma-associated antigen 59 with poly (lactic-co-glycolic acid): a promising nanovaccine candidate against *Haemonchus contortus* infection. Vet Parasitol. (2021) 292:109398. doi: 10.1016/j.vetpar.2021.109398, PMID: 33677347

[16] Kandeel M Akhtar T Zaheer T Ahmad S Ashraf U Omar M. Anti-parasitic applications of nanoparticles: a review. Pak Vet J. (2022):42, 2074–7764. doi: 10.29261/pakvetj/2022.040

[17] Ur-Rehman T El-Mansi AA Alhag SK Al-Shuraym LA Saeed Z Arif M. Antiparasitic activity of methanolic and ethyl acetate extracts of *Azadirachta indica* against *Haemonchus contortus*. Pak Vet J. (2023) 43:199–203. doi: 10.29261/pakvetj/2023.014

[18] Al-Saeed FA Ismael Bamarni SS Iqbal KJ Faruk AZ Mahmood S Şahin T. In vitro anthelmintic efficacy of Haloxylon salicornicum leaves extract using adult Heamonchus contortus Worms. Pak Vet J. (2023):43, 91–96. doi: 10.29261/pakvetj/2022.091

[19] Velázquez-Antunez J Olivares-Perez J Olmedo-Juárez A Rojas-Hernandez S Villa-Mancera A Romero RT. Biological activity of the secondary compounds of *Guazuma ulmifolia* leaves to inhibit the hatching of eggs of *Haemonchus contortus*. Pak Vet J. (2023):43:55. doi: 10.29261/pakvetj/2022.075

[20] Pakistan Economic Survey (2023). Government of Pakistan, finance division, economic advisor’s wing Islamabad. Available at: http://www.finance.gov.pk/survey_1819.html

[21] Troell K Engström A Morrison DA Mattsson JG Höglund J. Global patterns reveal strong population structure in *Haemonchus contortus*, a nematode parasite of domesticated ruminants. Int J Parasitol. (2006) 36:1305–16. doi: 10.1016/j.ijpara.2006.06.015, PMID: 16950266

[22] Yin F Gasser RB Li F Bao M Huang W Zou F. Genetic variability within and among *Haemonchus contortus* isolates from goats and sheep in China. Parasit Vectors. (2013) 6:1–9. doi: 10.1186/1756-3305-6-279, PMID: 24499637 PMC3852563

[23] Dey AR Zhang Z Begum N Alim MA Hu M Alam MZ. Genetic diversity patterns of *Haemonchus contortus* isolated from sheep and goats in Bangladesh. Infect Genet Evol. (2019) 68:177–84. doi: 10.1016/j.meegid.2018.12.021 30576839

[24] Gharamah AA Azizah MNS Rahman WA. Genetic variation of *Haemonchus contortus* (Trichostrongylidae) in sheep and goats from Malaysia and Yemen. Vet Parasitol. (2012) 188:268–76. doi: 10.1016/j.vetpar.2012.04.003, PMID: 22538095

[25] Ali M Majid HA Khan MR Muhammad D Ullah F Shuaib M. (2022). Prevalence and drug efficacy against gastrointestinal nematodes particularly *Haemonchus contortus* in district Kohat, Pakistan. World Journal of Pharmacy and pharmaceutical sciences, 11:107–115.

[26] Ruhoollah KW Al-Jabr OA Khan T Khan A El-Ghareeb WR Aguilar-Marcelino L. Prevalence of gastrointestinal parasite in small ruminants of district Dir upper Khyber Pakhtunkhwa Province of Pakistan. Braz J Biol. (2021) 83:e248978. doi: 10.1590/1519-6984.248978, PMID: 34669799

[27] Bibi R Afshan K Khan IA Iqbal Z Kayani AR Mushtaq M. Phenotyping and prevalence of *Haemonchus contortus* (Nematoda: Trichostongylidae) in ruminants from endemic areas of Pakistan: influence of host species and geographical area on phenotypic traits of Worms. Pak Vet J. (2017) 37:170–4.

[28] Qasim HM Avais M Durrani AZ Khan MA Shahzad AH. Dynamic dispersal of haemonchosis, its treatment and effect on blood profile of small ruminants of Lodhran district, Punjab, Pakistan. Pak J Zool. (2016) 48:755–61.

[29] Jamal Q Jafar S Shah A. Prevalence of *Haemonchus contortus* in markhor of chitral gol national park. J Sci Technol Univ Peshawar. (2016) 40:19–23.

[30] Jamil M Mansoor M Latif N Hussain N. Infection rate and chemotherapy of various helminths in *Ovis aries* in Dera Ismail Khan KPK, Pakistan. Int J. (2016) 2:7–17.

[31] Lashari MH Tasawar Z. Prevalence of some gastrointestinal parasites in sheep in southern Punjab, Pakistan. Pak Vet J. (2011) 31:295–8.

[32] Raza MA Younas M Schlecht E. Prevalence of gastrointestinal helminths in pastoral sheep and goat flocks in the Cholistan desert of Pakistan. J Anim Plant Sci. (2014):24, 127–134.

[33] Khalid M Muhammad I Durrani AZ Khan MA Sabir AJ Saleem MH. Infection rate and therapeutic trials on various gastrointestinal parasites in sheep and goats in and around Lahore, Pakistan. Pak J Zool. (2013) 45:489–94.

[34] Razzaq A Ashraf K Maqbool A Khan MA Islam M Khan H. Epidemiology, serodiagnosis and therapeutic studies on ovine nematodes at district Loralai, Balochistan, Pakistan. J Anim Plant Sci. (2013) 23:–1559.

[35] Ayaz MM Raza MA Murtaza S Akhtar S. Epidemiological survey of helminths of goats in southern Punjab, Pakistan. Trop Biomed. (2013) 30:62–71. PMID: 23665709

[36] Ullah N Khan MS Shah M. Infestation of helminthes parasite in sheep, *Ovis aries* (L.) in district Peshawar, Pakistan. Int. J. Biosci. (2013) 3:28–34. doi: 10.12692/ijb/3.2.28-34

[37] Akhter N Arijo A Phulan M Iqbal Z Mirbahar K. Prevalence of gastro-intestinal nematodes in goats in Hyderabad and adjoining areas. Pak Vet J. (2011) 31:287–90.

[38] Tasawar Z Ahmad S Lashari MH Chaudhary SH. Prevalence of *Haemonchus contortus* in sheep at research Centre for conservation of Sahiwal cattle (RCCSC) Jehangirabad District Khanewal, Punjab, Pakistan. Pak J Zool. (2010) 42:735–9.

[39] Khan MN Sajid MS Khan MK Iqbal Z Hussain A. Gastrointestinal helminthiasis: prevalence and associated determinants in domestic ruminants of district Toba Tek Singh, Punjab, Pakistan. Parasitol Res. (2010) 107:787–94. doi: 10.1007/s00436-010-1931-x, PMID: 20532913

[40] Gadahi J Arshed M Ali Q Javaid S Shah S. Prevalence of gastrointestinal parasites of sheep and goat in and around Rawalpindi and Islamabad, Pakistan. Vet World. (2009) 2:51–3.

[41] Raza MA Murtaza S Bachaya HA Dastager G Hussain A. Point prevalence of haemonchosis in sheep and goats slaughtered at Multan abattoir. J Anim Plant Sci. (2009) 28:158–9.

[42] Ijaz M Khan MS Avais M Ashraf K Ali MM. Infection rate and chemotherapy of various helminths in goats in and around Lahore. Pak Vet J. (2008) 28:167.

[43] Lateef M Iqbal Z Jabbar A Khan M Akhtar M. Epidemiology of trichostrongylid nematode infections in sheep under traditional husbandry system in Pakistan. Int J Agric Biol. (2005) 20:596–600.

[44] Jabeen F Ahmad N Ahmad KM Chaudhry MA Ali S. Studies on the epidemiology and chemotherapy of haemonchosis in sheep in the Punjab. Pak Vet J. (2000) 20:90–92.

[45] Sajid A Khan MQ Qayyum M Khan MFU. Prevalence of gastrointestinal parasites in sheep and goats maintained at NARC, Islamabad. Pak Vet J. (2000) 20:157–8.

[46] Jurasek ME Bishop-Stewart JK Storey BE Kaplan RM Kent ML. Modification and further evaluation of a fluorescein-labeled peanut agglutinin test for identification of *Haemonchus contortus* eggs. Vet Parasitol. (2010) 169:209–13. doi: 10.1016/j.vetpar.2009.12.003 20060646

[47] Ebrahim ZK. Effect of gastrointestinal parasites infestation on some hematological and biochemical parameters in sheep. Alex J Vet Sci. (2018):59, 44–47. doi: 10.5455/ajvs.1922

[48] Saitou N Nei M. The neighbor-joining method: a new method for reconstructing phylogenetic trees. Mol Biol Evol. (1987) 4:406–25. PMID: 3447015 10.1093/oxfordjournals.molbev.a040454

[49] Felsenstein J. Confidence limits on phylogenies: an approach using the bootstrap. Evolution (N Y). (1985) 39:783–91. doi: 10.1111/j.1558-5646.1985.tb00420.x 28561359

[50] Tamura K Nei M Kumar S. Prospects for inferring very large phylogenies by using the neighbor-joining method. Proc Natl Acad Sci. (2004) 101:11030–5. doi: 10.1073/pnas.0404206101, PMID: 15258291 PMC491989

[51] Tamura K Stecher G Kumar S. MEGA11: molecular evolutionary genetics analysis version 11. Mol Biol Evol. (2021) 38:3022–7. doi: 10.1093/molbev/msab120, PMID: 33892491 PMC8233496

[52] Kalule F Vudriko P Nanteza A Ekiri AB Alafiatayo R Betts J. Prevalence of gastrointestinal parasites and molecular identification of beta-tubulin mutations associated with benzimidazole resistance in *Haemonchus contortus* in goats from selected districts of Uganda. Vet Parasitol Reg Stud Reports. (2023) 42:100889. doi: 10.1016/j.vprsr.2023.100889 37321794

[53] Mussa SM. Study on the prevalence and associated risk factors of *Haemonchus contortus* infection in small ruminants in Mitto District, Silte zone, Ethiopia. J Vet Heal Sci. (2023) 4:46–53. doi: 10.33140/JVHS

[54] Gareh A Elhawary NM Tahoun A Ramez AM El-Shewehy DMM Elbaz E. Epidemiological, morphological, and morphometric study on Haemonchus spp. recovered from goats in Egypt. Front Vet Sci. (2021) 8:705619. doi: 10.3389/fvets.2021.705619, PMID: 34765663 PMC8575731

[55] Rinaldi L Catalan D Musella V Cecconi L Hertzberg H Torgerson PR. *Haemonchus contortus*: spatial risk distribution for infection in sheep in Europe. Geospat Health. (2015) 9:325–31. doi: 10.4081/gh.2015.355, PMID: 25826314

[56] Mushonga B Habumugisha D Kandiwa E Madzingira O Samkange A Segwagwe BE. Prevalence of *Haemonchus contortus* infections in sheep and goats in Nyagatare District, Rwanda. J Vet Med. (2018) 2018:1–9. doi: 10.1155/2018/3602081, PMID: 30271791 PMC6146870

[57] Besier RB Kahn LP Sargison ND Van Wyk JA. The pathophysiology, ecology and epidemiology of *Haemonchus contortus* infection in small ruminants. Adv Parasitol. (2016) 93:95–143. doi: 10.1016/bs.apar.2016.02.022, PMID: 27238004

[58] Akkari H Jebali J Gharbi M Mhadhbi M Awadi S Darghouth MA. Epidemiological study of sympatric Haemonchus species and genetic characterization of *Haemonchus contortus* in domestic ruminants in Tunisia. Vet Parasitol. (2013) 193:118–25. doi: 10.1016/j.vetpar.2012.12.014, PMID: 23333137

[59] Brik K Hassouni T Elkharrim K Belghyti D. A survey of *Haemonchus contortus* parasite of sheep from Gharb plain, Morocco. Parasite Epidemiol Control. (2019) 4:e00094. doi: 10.1016/j.parepi.2019.e00094, PMID: 30847410 PMC6378848

[60] Nabi H Saeed K Shah SR Rashid MI Akbar H Shehzad W. Epidimiological study of gastrointestinal nematodes of goats in district swat, Khyber Pakhtunkhwa, Pakistan. Sci. Int. (2014) 26:283–6.

[61] Urquhart GM Armour J Duncan JL Dunn AM and, Jennings FW. 2nd Veterinary Parasitology, Blackwell Science Ltd. Oxford (1996).

[62] Dorny P Symoens C Jalila A Vercruysse J Sani R. Strongyle infections in sheep and goats under the traditional husbandry system in peninsular Malaysia. Vet Parasitol. (1995) 56:121–36. PMID: 7732637 10.1016/0304-4017(94)00657-x

[63] ACM F Rajapakse R. Prevalence of coccidia and gastrointestinal nematode infections in cross bred goats in the dry areas of Sri Lanka. Small Rumin Res. (2001) 40:233–8. doi: 10.1016/S0921-4488(01)00179-1, PMID: 11323207

[64] Horak IG. Parasites of domestic and wild animals in South Africa. XLII. Helminths of sheep on four farms in the eastern Cape Province. Onderstepoort J Vet Res. (2003) 70:175–86. 14621313

[65] Adduci I Sajovitz F Hinney B Lichtmannsperger K Joachim A Wittek T. Haemonchosis in sheep and goats, control strategies and development of vaccines against *Haemonchus contortus*. Animals. (2022) 12:2339. doi: 10.3390/ani12182339, PMID: 36139199 PMC9495197

[66] Keyyu JD Kyvsgaard NC Monrad J Kassuku AA. Epidemiology of gastrointestinal nematodes in cattle on traditional, small-scale dairy and large-scale dairy farms in Iringa district, Tanzania. Vet Parasitol. (2005) 127:285–94. doi: 10.1016/j.vetpar.2004.10.014, PMID: 15710529

[67] Khajuria JK Kapoor PR. Prevalence of parasites in sheep and goats at Kathua-Jammu. J Vet Parasitol. (2003) 17:121–6.

[68] Nwosu CO Madu PP Richards WS. Prevalence and seasonal changes in the population of gastrointestinal nematodes of small ruminants in the semi-arid zone of North-Eastern Nigeria. Vet Parasitol. (2007) 144:118–24. doi: 10.1016/j.vetpar.2006.09.004 17127006

[69] Qamar MF Maqbool A Khan MS Ahmad N Muneer MA. Epidemiology of Haemonchosis in sheep and goats under different managemental conditions. Vet World. (2009) 2:413–7.

[70] Durrani Z Kamal N Khan S. Sero-diagnosis of Haemonchosis in small ruminants. Global Vet. (2007) 1:1–66.

[71] Rizvi AR Magrey TW Zia EUH. Clinical epidemiology and chemotherapy of haemonchosis in goats in Faisalabad. Pak. Vet. J. (1999) 54:107–9.

[72] Ouattara L Dorchies P. Gastro-intestinal helminths of sheep and goats in subhumid and sahelian areas of Burkina Faso. Rev. Med. Vet. (2001) 152:165–70.

[73] Hosseinnezhad H Sharifdini M Ashrafi K Atrkar Roushan Z Mirjalali H Rahmati B. Trichostrongyloid nematodes in ruminants of northern Iran: prevalence and molecular analysis. BMC Vet Res. (2021) 17:371. doi: 10.1186/s12917-021-03086-3, PMID: 34863161 PMC8642945

[74] Qamar W Zaman MA Faheem M Ahmed I Ali K Qamar MF. Molecular confirmation and genetic characterization of *Haemonchus contortus* isolates at the nuclear ribosomal ITS2 region: first update from Jhang region of Pakistan. Pak Vet J. (2022) 42:251–5. doi: 10.29261/PAKVETJ/2021.071

[75] Knoll S Dessì G Tamponi C Meloni L Cavallo L Mehmood N. Practical guide for microscopic identification of infectious gastrointestinal nematode larvae in sheep from Sardinia, Italy, backed by molecular analysis. Parasit Vectors. (2021) 14:505. doi: 10.1186/s13071-021-05013-9 34583765 PMC8477562

[76] Troell K. Genotypic and phenotypic characterization of *Haemonchus contortus* in Sweden. Uppsala: Department of Biomedical Sciences and Veterinary Public Health, Swedish University of Agricultural Sciences (2006).

